# Plume-subduction interaction forms large auriferous provinces

**DOI:** 10.1038/s41467-017-00821-z

**Published:** 2017-10-10

**Authors:** Santiago Tassara, José M. González-Jiménez, Martin Reich, Manuel E. Schilling, Diego Morata, Graham Begg, Edward Saunders, William L. Griffin, Suzanne Y. O’Reilly, Michel Grégoire, Fernando Barra, Alexandre Corgne

**Affiliations:** 1 Department of Geology and Andean Geothermal Center of Excellence (CEGA), FCFM, Universidad de Chile, Plaza Ercilla 803, Santiago, 8370450 Chile; 2 Departamento de Mineralogía y Petrología, Facultad de Ciencias, Universidad de Granada, Fuentenueva s/n, 180002 Granada, Spain; 3 Instituto de Ciencias de la Tierra, Facultad de Ciencias, Universidad Austral de Chile, Valdivia, 5090000 Chile; 4 Minerals Targeting International PL, 17 Prowse Street, West Perth, WA 6005 Australia; 5 Division of Earth Sciences, School of Environmental and Rural Science, University of New England, Armidale, NSW 2351 Australia; 6 ARC Centre of Excellence for Core to Crust Fluid Systems/GEMOC, Macquarie University, Sydney, NSW 2109 Australia; 7 GET, CNRS-CNES-IRD-UPS, Toulouse University, 14 Avenue Edouard Belin, 31200 Toulouse, France

## Abstract

Gold enrichment at the crustal or mantle source has been proposed as a key ingredient in the production of giant gold deposits and districts. However, the lithospheric-scale processes controlling gold endowment in a given metallogenic province remain unclear. Here we provide the first direct evidence of native gold in the mantle beneath the Deseado Massif in Patagonia that links an enriched mantle source to the occurrence of a large auriferous province in the overlying crust. A precursor stage of mantle refertilisation by plume-derived melts generated a gold-rich mantle source during the Early Jurassic. The interplay of this enriched mantle domain and subduction-related fluids released during the Middle-Late Jurassic resulted in optimal conditions to produce the ore-forming magmas that generated the gold deposits. Our study highlights that refertilisation of the subcontinental lithospheric mantle is a key factor in forming large metallogenic provinces in the Earth’s crust, thus providing an alternative view to current crust-related enrichment models.

## Abstract (web summary)

The lithospheric controls on giant gold deposits remain unclear. Here, the authors show evidence for native gold in the mantle from the Deseado Massif in Patagonia demonstrating that refertilisation of the lithospheric mantle is key in forming metallogenic provinces.

## Introduction

The traditional notion of Au endowment in a given metallogenic province is that Au accumulates by highly efficient magmatic-hydrothermal enrichment processes operating in a chemically ‘average’ crust. However, more recent views point to anomalously enriched source regions and/or melts that are critical for the formation of Au provinces at a lithospheric scale^1–4^. Within this perspective, Au-rich melts/fluids might originate from a mid or lower crust reservoir and later migrate through favourable structural zones to shallower crustal levels where the Au deposits form^5^. Alternatively, the subcontinental lithospheric mantle (SCLM) may also play a role as a source of metal-rich magmas^2, 3, 6, 7^. This model involves deep-seated Au-rich magmas that may infiltrate the edges of buoyant and rigid domains in the SCLM producing transient Au storage zones. Upon melting, the ascending magma scavenges the Au as it migrates towards the uppermost overlying crust^6, 8^. Discontinuities between buoyant and rigid domains in the SCLM provide the channelways for the uprising of Au-rich fluids or melts from the convecting underlying mantle, and when connected to the overlying crust by trans-lithospheric faults, a large Au deposit or well-endowed auriferous province can be formed^7^. Thus, the generation of Au deposits in the crust may result from the conjunction in time and space of three essential factors: an upper mantle or lower crustal source region particularly enriched in Au, a transient remobilisation event and favourable lithospheric-scale plumbing structures. The giant Ladolam Au deposit in Papua New Guinea gives a good single-deposit case example of this mechanism since deep trans-lithospheric faults connect the crustal Au deposit directly with the mantle source, and similar Os isotopic compositions are exhibited by Au ores and metal-enriched peridotite of the underlying mantle^1^. Despite these evidences, the genetic relation between a pre-enriched mantle source and the occurrence of gold provinces in the upper crust remains controversial since limited evidence is available at a broader regional scale.

In this paper, we provide the first empirical evidence connecting the genesis of a large Au province (Deseado Massif, Argentina ~15 Moz Au, ~400 Moz Ag^2, 9, 10^, see additional references in Supplementary Information) to the occurrence of ‘visible’ Au in the underlying SCLM. Our observations of ultramafic xenoliths sampled from monogenetic volcanoes from the Deseado Massif, southern Patagonia, provide unprecedented evidence that an enriched SCLM might be the primary source for the generation of this auriferous province.

## Results

### Geological setting and xenolith petrology

**Fig. 1:**
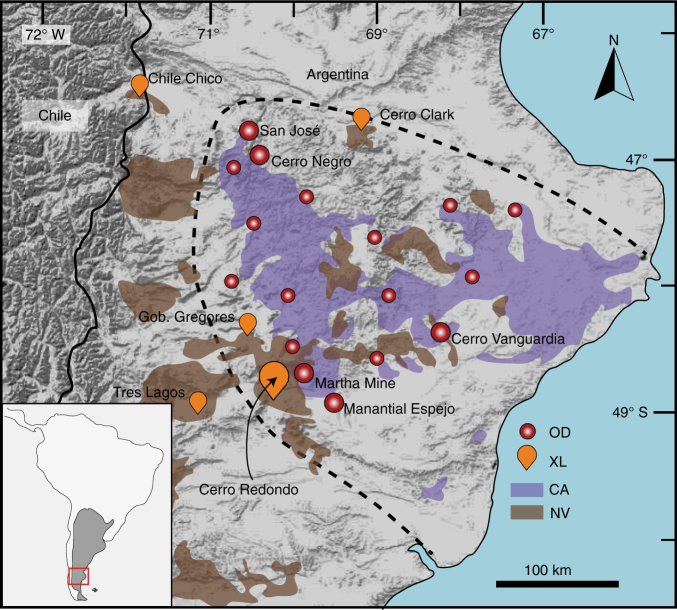
Simplified geological map of southern Patagonia Argentina. The *dashed line* delimits the Deseado Massif auriferous province. CA, Chon Aike volcanic sequences; NV, Neogene volcanism; OD, most relevant ore deposits and prospects; XL, location of different xenolith sites in the Deseado Massif and surroundings

The Deseado Massif is an underexplored auriferous province of ~60,000 km^2^ located in the southernmost part of Argentina in South America^9^. It hosts several Au–Ag epithermal deposits including low sulfidation, intermediate sulfidation and polymetallic epithermal deposits associated with calc-alkaline rhyolites, basaltic andesites and basalts from the late magmatic stages of the Chon Aike silicic large igneous province (CA-SLIP)^11, 12^ **(**Fig. 1). The CA-SLIP is represented by the extensive volcanism that was active from 187 to 144 Ma contemporaneously with the initial break-up of Gondwana^13^, and includes two main stages of petrogenesis. The Early Jurassic magmatic pulses of the CA-SLIP are ascribed to crustal melting caused by spreading of the Karoo plume head (~180 Ma), whereas the geochemical signature of Middle to Late Jurassic events show the influence of an active subduction margin in a back-arc position (~155 Ma). The latter is coincident to the migration of magmatism away from the Karoo mantle plume towards the proto-Pacific margin of Gondwana during rifting and break-up^14, 15^. An extensive Neogene back-arc plateau magmatism composed of alkaline basalts (~3.5 Ma) has brought to the surface deep-seated mantle xenoliths from beneath the crust that host the Au mineralisation^16–22^. Dominantly spinel lherzolites of the Cr-diopside suite^23^, these xenoliths record Meso to Paleoproterozoic partial melting^20–22^ and subsequent multistage modification of the mantle, including carbonatitic, silica-undersaturated alkaline and subduction-related metasomatism^17–19^.

**Fig. 2:**
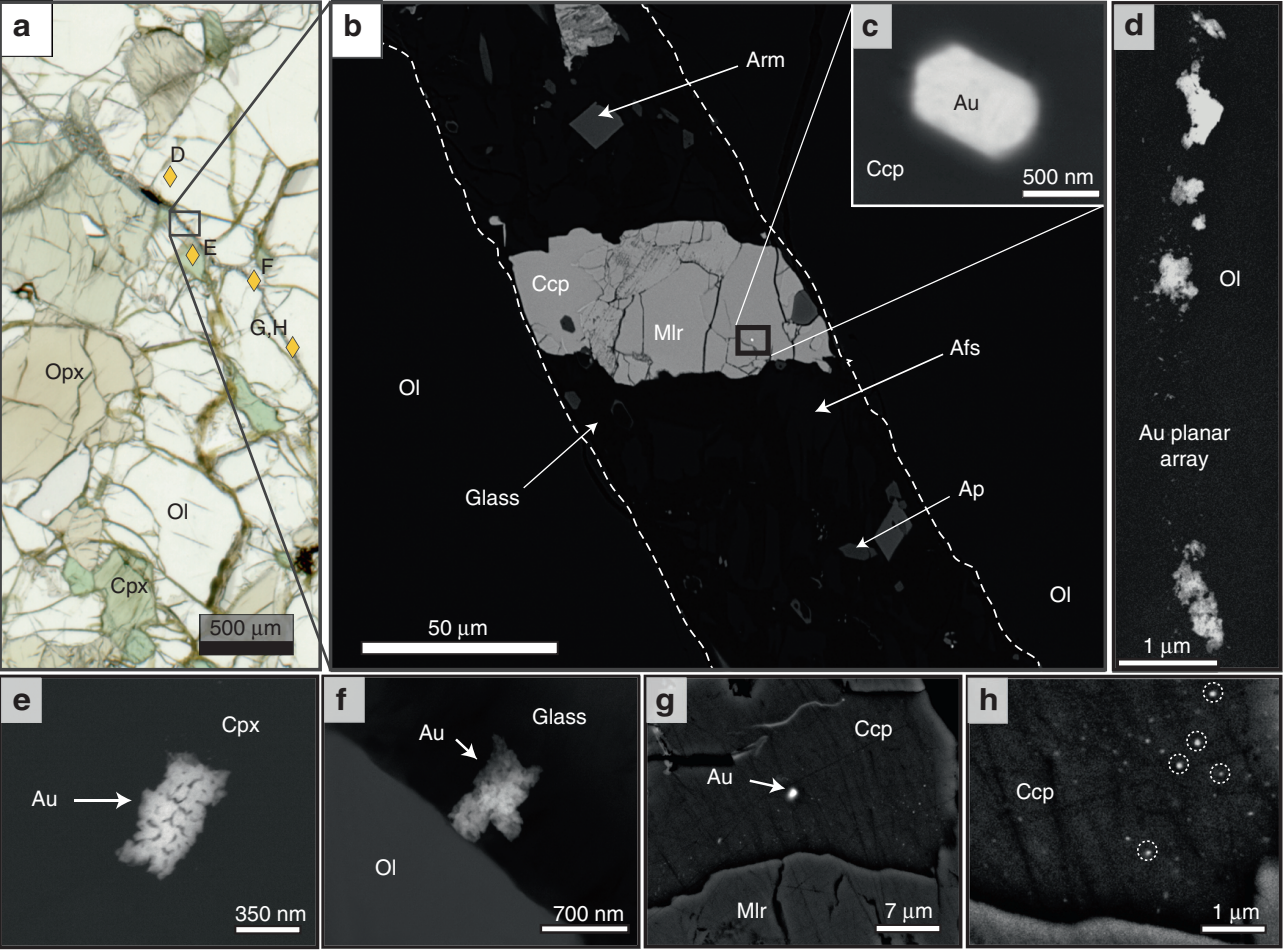
Photomicrograph and backscattered electron (BSE) images of Au particles in the Cerro Redondo mantle xenolith. **a** Plane polarised light image of the lherzolite sample showing the late metasomatic glass vein and the location of Au particles (*golden diamonds* and *letters* refer to BSE images). **b**–**h** Backscattered electron FE-SEM images of Au particles and their microstructural setting. **b** Detail of the glass vein showing its metasomatic assemblage and a composite sulfide grain containing a Au particle. **c** Magnification of the euhedral Au particle within chalcopyrite from the composite sulfide grain in **b**. **d** Planar array of Au particles within olivine. **e** Au particle enclosed within clinopyroxene. **f** Au particle within the glass of the metasomatic vein in contact with olivine. **g** Au particle within chalcopyrite and arrangement of Au nanoparticles enlarged in **h**. Afs, alkali feldspar; Ap, apatite; Arm, armalcolite; Ccp, chalcopyrite; Cpx, clinopyroxene; Mlr, millerite; Ol, olivine; Opx, orthopyroxene

The Au-bearing ultramafic xenolith studied here was collected from the Cerro Redondo cinder cone, located at the south-western edge of the Deseado Massif (49°7′15.41″ S; 70°8′28.56″ W), and was chosen as a case study since it samples the mantle directly beneath the Au–Ag deposits of ‘Manantial Espejo’, and ‘La Rosita’ and ‘La Sarita’ Au–Ag prospects (Fig. 1). The targeted xenolith is a protogranular anhydrous mantle lherzolite that equilibrated in the spinel facies (up to 1.76 GPa, ca. ~53 km depth) at temperatures of 1020–1150 °C (Supplementary Table 1). The lherzolite xenolith records at least three stages of chemical depletion and enrichment: Stage I comprises the formation of a chemically depleted residue after the removal of ~5–10% partial melt, which is recorded in primary olivine with very low Al_2_O_3_ and CaO and Mg# = 90.1–91.1 (Supplementary Data 1) and slightly depleted light rare earth elements/ heavy rare earth elements (LREE/HREE) ratios in clinopyroxene. Stage II involved melt infiltration and precipitation of metasomatic pyroxene with high Al_2_O_3_ and TiO_2_ contents and LREE enrichment from silicate alkaline to sub-alkaline melts (Supplementary Data 2 and Supplementary Fig. 1). A third event (Stage III) is evidenced by the infiltration of metasomatic melts just before the xenolith was entrained into the Neogene basalt, which resulted in the formation of interstitial silicate glass containing native Au particles. This interstitial glass is unrelated to the host basalt and is partially altered to secondary clays that overprint the entire sample. The unaltered mineral assemblage in this glass consists of incompletely reacted olivine and pyroxenes together with armalcolite [(Mg,Fe^2+^)Ti_2_O_5_], ilmenite, feldspar and apatite (Supplementary Data 1). Composite aggregates (10–120 μm) of chalcopyrite, pentlandite and millerite are common within the glass, and to a lesser extent within the primary silicates (Fig. 2a, b) (Supplementary Table 2).

### Au entrained by infiltrating melts

Gold particles (<2 µm) were found enclosed within primary olivine and pyroxene (Fig. 2d, e) and embedded in the glass matrix (Fig. 2f) or sulfides (Fig. 2b, c, g, h) in the interstitial glass. Energy dispersive X-ray spectra (EDS) obtained using a high-resolution, field-emission scanning electron microscope (FE-SEM) and filtered for host matrix composition (Supplementary Figs. 3–8) indicate that all these grains are almost pure Au. Gold grains found in primary silicates and in the groundmass of the interstitial glass are small (<750 nm across), irregular and commonly aligned forming planar arrays within the silicate host (Fig. 2d). In contrast, Au particles located inside larger sulfide grains, mainly chalcopyrite, embedded in the interstitial glass are relatively larger (~1.5 µm) and display well-developed polygonal faces of the cubic crystallographic system. Detailed inspection of the chalcopyrite hosts reveals abundant native Au nanoparticles (Fig. 2g, h), which is consistent with the high Au (up to 6 p.p.m.) obtained by laser ablation-inductively coupled plasma-mass spectrometry (LA-ICP-MS) analysis of these sulfides. It is relevant to note that these sulfides contain significant amounts of Ag (up to 163 p.p.m., Supplementary Table 3). The Au/Ag ratios of the mantle sulfides are similar to the Au/Ag ratios of the bulk ores, and may exert and important control on the economic metal ratios of epithermal Au–Ag deposits of the Deseado Massif (Au/Ag ~0.01–1, Supplementary Fig. 2).

The occurrence of native Au particles forming planar arrays within olivine or pyroxene suggests a physical entrapment mechanism, most likely facilitated by structural discontinuities (e.g., cleavage planes or zones for local accumulation of dislocations) during magmatic silicate growth. These native Au particles are free of any secondary hydrothermal markers such as Pb, Ag, Te, Bi, Cu and Sb^24, 25^ ruling out the possibility that these Au particles were included within olivine during secondary subsolidus growth as observed in peridotites of the Lherz massif (France)^26^. Furthermore, the estimated temperatures for the equilibration of primary silicates in the studied xenolith exceeded >1000 °C, which precludes an origin related to secondary silicate growth.

The fact that Au is included in pyroxene and olivine that crystallised at different stages of the depletion–refertilisation history of the SCLM and at a different time scale also excludes the possibility that Au found in these silicates crystallised contemporaneously from the same parental melt. Instead, the fact that Au particles in primary silicates are only found in those grains that are in close proximity (<500 μm) to the interstitial glass also containing Au particles more likely indicates that Au was introduced later by an infiltrating melt now quenched as a glassy vein. Deposition of Au by the infiltrating melt could have occurred through discontinuities or microsized crack seals, which would be later sealed during silicate annealing. Crack propagation is a well-known mechanism for the almost instantaneous movement of fluids in the mantle^27^, and it can occur due to fluid overpressure or where crystallisation in pore space in subsolidus matrices is more rapid than the viscous relaxation of the rock.

## Discussion

Experimental studies have reported the formation of metallic Au-rich alloys at magmatic temperatures (>1200 °C) from S-undersaturated and Fe-containing silicate melts, under either reducing conditions buffered by iron wustite (IW)^28^ or at *f*O_2_ near the fayalite–magnetite–quartz (FMQ) buffer^29^. The results of these experiments demonstrate that native Au can crystallise over a wide range of *f*O_2_ conditions depending on the sulfur content in the basaltic melt. In fact, native Au micronuggets have been documented in the glassy groundmass of mantle-derived rocks such as lamproite dykes segregated at *f*O_2_ near the IW buffer^30^ and basanite lavas from Hawaii formed at ΔFMQ ≈+0.4^31^. The former author linked the direct crystallisation of native Au from the lamproite magma to strongly reducing conditions, whereas the latter argued for the solidification of droplets of immiscible Au liquids. In the latter, an increase in alkali contents upon fractionation of the melt increased the solubility of sulfur, which promoted the resorption of sulfides entrained in the melt and the segregation of immiscible Au liquid droplets.

The Au-bearing interstitial glass from the Cerro Redondo mantle xenolith contains a mineral assemblage that includes armalcolite, ilmenite, feldspar, apatite and Au-bearing sulfides, reflecting the crystallisation of alkalic melts unusually enriched in incompatible elements^32, 33^ and slightly more reduced (ΔFMQ ≈ −2.35 at 1150–1200 °C and 1.3 GPa^33^) than estimated for the primary silicates (ΔFMQ ≈ 0.12–1.06; Supplementary Table 4). These *f*O_2_ values reflect the infiltration of the alkalic melt into a slightly more oxidised mantle peridotite. The idea that Au could be transported by sulfide melts entrained in the silicate melt^34^ is supported by the fact that clusters of native Au nanoparticles and euhedral Au inclusions are found hosted in Cu-rich sulfides (Fig. 2). Upon ascent, the infiltrating melt should undergo decompression, as well as fractionation and oxidation by reaction with the country rocks. The combination of these processes may have shifted the balance between the stability of sulfide towards the more soluble sulfate field^35–37^, promoting sulfide resorption as evidenced by the presence of oxide rims in some of the studied grains (Supplementary Fig. 8). This would result in the liberation of Au as discrete grains from the sulfides with relatively high contents of Au (Fig. 2, Supplementary Table 3). Subsequent quenching of the melt into a glass would prevent complete dissolution of Au particles, whereas injection of some of them in primary silicates would prevent further reaction with the infiltrating silicate melt(s). These Au-bearing sulfides would undergo further closed-system subsolidus modification during transport or after solidification of the host silicate melt, promoting decomposition of the original monosulfide solid solution into Ni-rich and Cu-rich sulfides.

As noted above, a late infiltrating melt, i.e., the glassy vein, introduced Au into the Cerro Redondo mantle peridotite. This Au-bearing glass vein could represent: host basalt that penetrated the xenolith, melt(s) produced by decompression melting of the mantle peridotite during its ascent to the surface or small volume melt(s) that infiltrated the mantle before the entrapment of the xenoliths by the host basalt. If the glass resulted from the injection of the enclosing basalt or from peridotite–host basalt interaction, the interstitial vein and enclosing basalt should have similar composition and mineral assemblages. Instead, the interstitial glass contains a very rare mineral assemblage made up of armalcolite, ilmenite, feldspar and apatite. This type of mineral assemblage has been related to the crystallisation of highly alkaline melts, anomalously enriched in incompatible elements^33^, with a significant chemical mismatch with the host basalt or eventual products of the reaction between the host basalt and the mantle xenolith. This exotic mineral assemblage could not have been formed by simple decompression melting owing the nature of the primary minerals forming the xenolith. Therefore, the Au-bearing interstitial glass observed in the studied xenolith most likely represents the quenched product of a distinctive melt that predates the eruption of the alkali basalts that brought the xenoliths to the surface ~3.5 Ma ago.

A similar metasomatic assemblage including armalcolite, ilmenite, feldspar and apatite was documented in peridotite xenoliths that have sampled the mantle underlying the plume-related hotspot of the Kerguelen Islands^32, 33^. Moreover, terrestrial occurrences of armalcolite, although not very rare, are mostly restricted to certain picritic lavas from the Karoo igneous province^38^, high TiO_2_ basalts from the Kilauea volcano, Hawaii^39^ and ultrapotassic lavas from southeastern Spain^40^. The first two are related to plume activity and the latter to a highly enriched lithospheric mantle. Furthermore, native Au particles have been reported in the Kilauea and southeastern Spain lavas^30, 31^. Therefore, we interpret that the Au-bearing highly alkaline melt might also represent the melting product of a mantle domain previously affected by infiltration of plume-related melts.

Interestingly, mantle plume activity has been suggested to produce large enrichments of Au (and noble metals) in the lithospheric mantle and related crust overlying the Iceland plume^4^. Further evidence for such type of enrichment includes a 30 µm bleb of pure Au enclosed in a fresh olivine phenocryst in a picritic lava from the Emeishan large igneous province in south-western China. This Au bleb was interpreted as a xenocryst of the deep mantle transported to shallow depths by a rising plume and then captured by picritic melts^41^. Rising plumes originating in the core-mantle boundary may potentially add Au into the SCLM during their final ascent^42^.

During the Jurassic, active volcanism took place in Patagonia in a geodynamical environment characterised by an extensional regime related to the initial stages of the break-up of Gondwana and the emplacement of the Karoo superplume to the east, and episodic subduction from southern America to Antarctica to the west^13, 43^. Interestingly, the epithermal Au–Ag deposits of the Deseado Massif auriferous province are related to the calc-alkaline stages of the CA-SLIP. Contrary to models of purely crustal origin, some authors have suggested that the bimodal CA-SLIP was produced from melts derived from a portion of SCLM that was metasomatised and later thermally eroded by the effects of the Karoo mantle plume^44–47^.

**Fig. 3:**
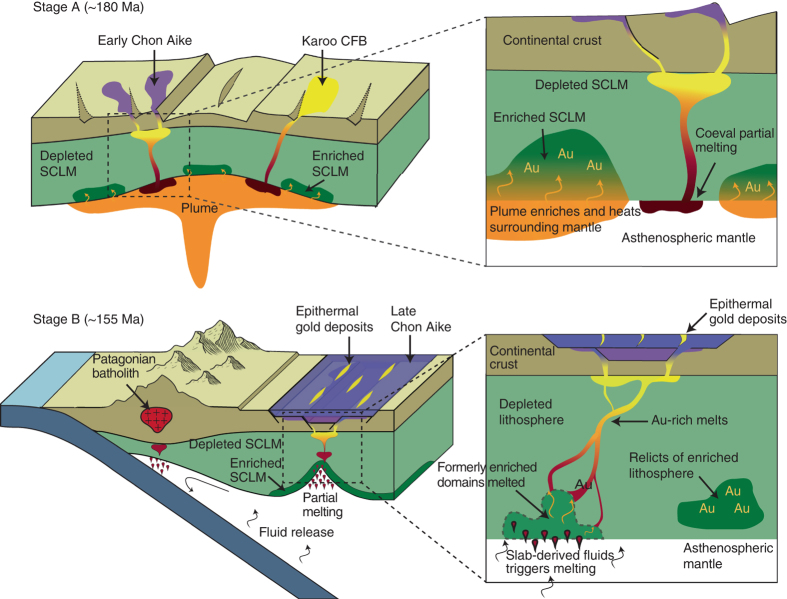
Lithospheric-scale processes involved in the precursor stage of formation of the Deseado Massif auriferous province. Stage A: plume activity during Early Jurassic related to the initial stages of Gondwana break-up induces metasomatic Au enrichment in the overlying SCLM and coeval partial melting. The *inset* shows the transfer of Au to the enriched domains and partial melting processes responsible for the early magmatic stages of the CA-SLIP. Stage B: onset of the subduction zone at the western margin of Gondwana provides fluids capable of scavenging Au from formerly enriched domains and generates calc-alkaline magmatism represented by the middle-late magmatic stages of the CA-SLIP that hosts the Au deposits. The *inset* shows the process of partial melting of enriched domains and Au transport to crustal levels; some portions of enriched lithosphere remain unmodified

Considering this scenario, infiltration of melts derived from the Karoo superplume could have added Au to the SCLM beneath the Patagonian auriferous province (Fig. 3a). It is likely that this mantle domain was affected by partial melting and already transferring metals to the overlying continental crust since at least the Proterozoic^21, 22^. The fact that the Au-bearing glassy vein in the studied xenolith was formed just before its exhumation in the Neogene, suggests that not all of the enriched SCLM was affected by the processes involved in the generation of the gold deposits of the Deseado Massif province during the Late Jurassic. This provides evidence that enriched domains of the SCLM might be relatively durable and store Au (and Ag) over large periods of time until a later melting event triggers the release of the stored metals^7^. As noted above, the Au-bearing metasomatic vein in the Cerro Redondo peridotite xenolith represents the re-melting of these mantle domains, highly enriched in both lithophile and siderophile incompatible elements.

Consequently, the Patagonian CA-SLIP and concomitant auriferous province is the result of mantle plume activity generated during extension in a back-arc setting while subduction of oceanic lithosphere occurred at the western margin of Gondwana^13, 14^ (Fig. 3b). Subduction was a necessary component to trigger mantle melting, as it lowered the peridotite solidus and contributed oxidised fluids capable of scavenging Au (i.e., ~FMQ>1). This is in good agreement with the fact that the Deseado Massif mineralisation is hosted by the calc-alkaline stages of the CA-SLIP.

The Deseado Massif auriferous province was the result of an optimal amalgamation of three main factors: (i) the influence of a mantle plume, which enriched a SCLM domain in incompatible elements (including Au) and provided the necessary heat to produce large volumes of magma over almost ~40 Ma during the Jurassic (Fig. 3a); (ii) the influence of the subduction zone setting at the western (Pacific) margin of Gondwana, which may have oxidised portions of the enriched mantle domain, enhancing the potential of magmas to transport Au as dissolved species (Fig. 3b); and (iii) an extensional geodynamic setting that facilitated these enriched magmas to ascend to crustal levels and form the epithermal Au–Ag deposits (Fig. 3b).

We argue for a genetic model involving processes operating at a lithospheric scale as the first-order factor controlling the early stages of the formation of an auriferous province. These processes may be overlapped in space but separated by large periods of time. Thus, understanding global tectonics and evolution of the SCLM through Earth**’**s history is of fundamental importance to understand the factors that control metal transfer from deep mantle sources to the uppermost crust, and is a critical step in the development of new strategies for successful gold exploration worldwide.

## Methods

### Field-emission scanning electron microscopy

All sulfides and gold particles were imaged using a JEOL JSM-7100 FE-SEM at the Serveis Cientificotècnics, University of Barcelona, Spain; and a QUANTA 650 FEG environmental SEM (E-SEM) at the Instituto Andaluz de Ciencias de la Tierra, University of Granada, Spain. Both the FE-SEM and E-SEM are equipped with SE, BSE and EDS detectors. Accelerating voltage was 20 kV and beam current optimised for a sufficient number of counts for each EDS analysis.

### Electron microprobe analyses

The major and minor element composition of silicates was determined using a FE Cameca SXFive electron microprobe at the Raimond Castaing Center, Toulouse University. The operating conditions were: accelerating voltage 15 kV; beam current 20 nA; and analysed surface is around 2 × 2 μm^2^. The following standards were used: albite (Na), periclase (Mg), corundum (Al), sanidine (K), wollastonite (Ca, Si), pyrophanite (Mn, Ti), haematite (Fe), Cr_2_O_3_ (Cr), NiO (Ni), sphalerite (Zn) and V metal (V).

Sulfide mineral chemical analyses were performed with a five-channel JEOL JXA-8230 electron microprobe at the Serveis Cientificotècnics, University of Barcelona, Spain. The operating conditions were: accelerating voltage 20 kV, beam current 20 nA and a 5 μm beam diameter. The following standards were used: pyrite (S, Fe), Ni metal (Ni), chalcopyrite (Cu), Co metal (Co) and sphalerite (Zn).

### Laser ablation-inductively coupled plasma-mass spectrometry

Concentrations of trace elements in clinopyroxene were determined in situ by LA-ICP-MS using a NewWave Research UP213 laser coupled to an Agilent 7500 ICP-MS instrument (Raimond Castaing Center, Université Paul Sabatier—Toulouse III, France). NIST 610 and NIST 612 glass standards were used to calibrate relative element sensitivities. Each analysis was normalised using the Ca content determined by electron microprobe. A beam diameter of 50 μm and a scanning rate of 20 μm s^−1^ was used. Typical theoretical detection limits range from 10 to 20 p.p.b. for all the elements analysed.

Trace element concentrations of sulfides were carried out in the geochemical analysis unit at CCFS/GEMOC, Macquarie University, Sydney using LA-ICP-MS. Helium was used as the carrier gas, which was blended with Ar prior to introduction into the plasma. The laser ablation system was operated at 5 Hz with an average beam energy 6.9 mJ per pulse. Sulfur, determined by EMP, was used as an internal standard for quantifying the trace element abundances. A quenched NiS (PGE-A: Alard et al.^48^ and Alard et al.^49^), doped with selected chalcophile and siderophile elements was used as an external calibration standard. Detection limits for LA-ICP-MS analyses are calculated as average background concentrations plus three standard deviations. Average detection limits for Au and PGE are: Au (0.008 p.p.m.); Os (0.08 p.p.m.); Ir (0.01 p.p.m.); Ru (0.03 p.p.m.); Rh (0.006 p.p.m.); Pt (0.03 p.p.m.) and Pd (0.04 p.p.m.).

### Data availability

The authors declare that the data supporting the findings of this study are available within the paper and its Supplementary Information files.

## Electronic supplementary material

Supplementary Information

Data Set 1

Data Set 2

Peer Review file
