# Supplementary material for: Plume-subduction interaction forms large auriferous provinces
Source: Nat Commun. 2017 Oct 10;8:843. doi: 10.1038/s41467-017-00821-z (PMC5634996; doi:10.1038/s41467-017-00821-z)
Supplement: Supplementary file 1 — Supplementary Information [file 41467_2017_821_MOESM1_ESM.pdf]

Description of Supplementary Files

File Name: Supplementary Information
Description: Supplementary Figures, Supplementary Tables and Supplementary References

File Name: Supplementary Data 1
Description: Major-element composition of primary and accessory minerals related to the late metasomatic vein. Electron microprobe analyses.

File Name: Supplementary Data 2
Description: REE and Trace element concentration of clinopyroxenes. All values are in ppm. LA-ICP-MS analyses.

File Name: Peer Review File

Pressure and Temperature conditions on the studied samples		
	**Equilibration Pressures (GPa)**	**Equilibration Temperatures (°C)**
**(Ref. 1)**	1.5	1,121
	1.5	1,113
	1.5	1,149
**(Ref. 2)**	1.3	1,068
	1.1	1,020
	1.7	1,114
**(Ref. 3)**	1.5	1,068
	1.5	1,020
	1.5	1,114

Supplementary Table 1. Summary of the thermobarometers used in the P-T estimation of the equilibrium conditions of the primary assemblage and their results.

Pressure and temperature were estimated using iterative calculations based on P-sensitive thermometers and T-sensitive barometers[1-3].

N°	Phase	S	Fe	Co	Ni	Cu	Zn	Total
1	Chalcopyrite	34.65	29.76	0.04	0.55	34.21	<DL	99.21
2	Chalcopyrite	34.88	29.26	0.05	0.85	34.13	0.02	99.64
3	Chalcopyrite	34.79	29.83	0.04	0.21	34.70	0.01	99.58
4	Chalcopyrite	34.48	29.48	0.04	0.53	34.66	0.02	99.21
5	Millerite	34.51	4.87	0.66	59.04	0.27	0.01	99.47
6	Millerite	35.34	2.77	0.36	61.58	0.17	<DL	100.50
7	Millerite	35.04	1.40	0.14	62.36	0.32	<DL	99.76
8	Millerite	35.13	1.38	0.14	62.27	0.32	0.03	99.74
9	MSS	33.02	24.14	0.30	42.09	0.14	<DL	99.68
10	MSS	32.66	14.97	0.18	50.20	0.59	<DL	98.60
11	Pentlandite	33.19	24.69	0.42	40.03	0.20	<DL	98.53
12	Pentlandite	33.23	26.99	0.56	39.09	0.03	<DL	99.91
13	Pentlandite	38.87	36.05	0.30	24.58	0.06	<DL	99.86
14	Pentlandite	33.14	27.14	0.63	39.23	0.09	<DL	100.22
15	Pentlandite	32.99	27.32	0.55	38.91	0.11	<DL	99.88
16	Pentlandite	33.20	26.62	0.56	39.09	0.06	<DL	99.53
17	Pentlandite	33.47	24.14	0.49	39.15	1.20	<DL	98.45

Supplementary Table 2. Major-element composition of sulphides embedded in the metasomatic late vein. <DL: below detection limit. All values are in wt.%.

N°	1	2	3	4	5	6	7	8	9
**Os**	1.83	81.54	281.99	2.20	2.95	83.44	25.22	27.47	23.51
**DL**	0.06	0.06	0.04	0.06	0.06	0.23	0.00	0.07	0.07
**Ir**	0.96	67.34	135.55	0.13	8.16	32.80	12.12	12.05	11.38
**DL**	0.01	0.01	0.00	0.01	0.00	0.03	0.01	0.01	0.01
**Ru**	0.48	33.43	72.06	2.51	13.70	32.27	12.49	12.44	12.10
**DL**	0.01	0.02	0.00	0.00	0.00	0.06	0.00	0.02	0.02
**Rh**	0.22	8.25	15.59	1.42	3.63	7.44	2.10	2.94	1.80
**DL**	0.00	0.00	0.00	0.00	0.00	0.01	0.00	0.00	0.00
**Pt**	14.07	258.85	3.66	0.00	0.88	31.33	2.18	0.30	0.06
**DL**	0.01	0.03	0.02	0.00	0.02	0.08	0.03	0.03	0.02
**Pd**	15.91	41.97	43.41	2.49	28.97	38.02	0.06	32.63	0.62
**DL**	0.01	0.01	0.00	0.01	0.00	0.05	0.02	0.02	0.01
**Au**	0.67	5.96	4.40	0.00	0.00	3.54	0.02	0.37	0.05
**DL**	0.01	0.01	0.00	0.01	0.00	0.02	0.01	0.01	0.01
**Ag**	0.70	23.09	162.96	49.31	42.90	64.24	0.10	3.22	2.44
**DL**	0.02	0.01	0.01	0.02	0.01	0.05	0.01	0.01	0.01
**∑PGE**	33.46	491.38	552.26	8.75	58.29	225.30	54.17	87.83	49.46
**Total**	34.83	520.43	719.62	58.06	101.19	293.08	54.28	91.42	51.95

Supplementary Table 3. Noble metal content of sulphides from Patagonian mantle xenoliths obtained by LA-ICP-MS. <DL: below detection Limit. All values are in ppm.

		A	B	C
**Olivine**	**XFe**	0.09	0.09	0.09
	**XMg**	0.91	0.91	0.91
**Orthopyroxene**	**XM1 (Fe)**	0.09	0.09	0.09
	**XM2 (Fe)**	0.08	0.09	0.08
**Spinel**	**Fe^{3+}**	0.04	0.06	0.08
	**Fe^{2+}**	0.19	0.22	0.19
	**log a(Fe_3O_4)**	-2.01	-1.84	-1.48
**Temperature (°C)**		1,020 1,149	1,020 1,149	1,020 1,149
**ΔFMQ**		-0.03 0.24	0.44 0.71	0.97 1.25

Supplementary Table 4. Summary of the data used in the oxygen fugacity conditions estimation for three different equilibrated assemblages (A, B and C) for the minimum and maximum temperatures estimated from the thermometers mentioned above.

The fO_2 conditions of the sample were calculated using the following reaction equilibrium:

$$\mathbf{6\ Fe_2SiO_4\ (in\ olivine) + O_2 = Fe_2Si_2O_6\ (in\ orthopyroxene) + 2\ Fe_3O_4\ (in\ spinel)} \qquad (1)$$

The Fe^{3+} contents in spinel was calculated from electron microprobe analyses assuming perfect stoichiometry. Thus, the Fe^{3+} content was converted to activities of magnetite (Fe_3O_4) in spinel using the formula below[4]:

$$\mathbf{\log a(Fe_3O_4\ in\ Sp) = \log ((Fe^{2+})*Fe^{3+})^2/4) + 1/T\ [406\ (Al)^2 + 653\ (Mg)*(Al) + 299\ (Cr)^2 + 199\ (Al)*(Cr) + 346\ (Mg)*(Cr)]} \qquad (2)$$

The terms in parentheses refer to total atomic concentrations of Mg, Fe^{2+}, Fe^{3+}, Cr and Al in spinel on a four oxygen basis. K is temperature in Kelvin.

Therefore, $\Delta \log fO_2$(FMQ) at a given pressure (in bar) and temperature (in K) is given by the following equation:

$$\mathbf{\Delta \log fO_2(FMQ) = \log (fO_2) - \log (fO_2)_{FMQ} = 220/T + 0.35 - 0.0369\ P/T - 12 \log X_{Fe}Ol - 2620(X_{Mg}Ol)^2/T + 3\log [X_{Fe}M1)Opx * (X_{Fe}M2)Opx] + 2 \log a(Fe_3O_4\ in\ Sp)} \qquad (3)$$

$\Delta \log fO_2$(FMQ) is fO_2 relative to FMQ buffer, P in bar, T in Kelvin, $X_{Fe}Ol$ and $X_{Mg}Ol$ refer to the atomic ratio of $Fe^{2+}/(Mg+Fe^{2+})$ and $Mg/(Mg+Fe^{2+})$ in olivine respectively. ($X_{Fe}M1$)Opx and ($X_{Fe}M2$)Opx refer to the atomic fractions of Fe in the M1 and M2 sites of orthopyroxene, and were calculated following the methods in ref. 5.

a(Fe_3O_4 in Sp) is activity of magnetite, Fe_3O_4, in spinel.

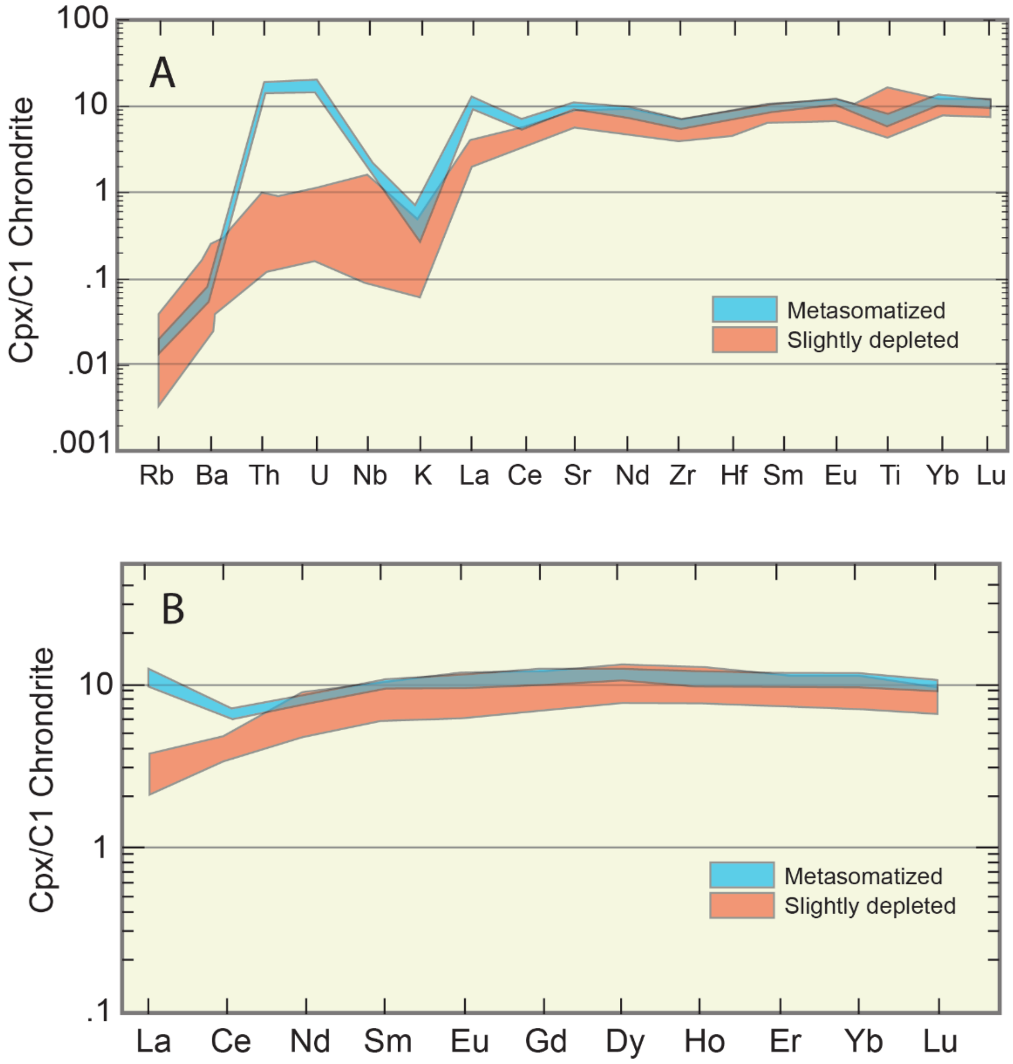

Supplementary Figure 1. Chondrite-normalized trace and REE patterns from clinopyroxenes from Cerro Redondo mantle xenolith reflecting at least two generations of the silicate primary assemblage. Normalizing values are from ref. 6.

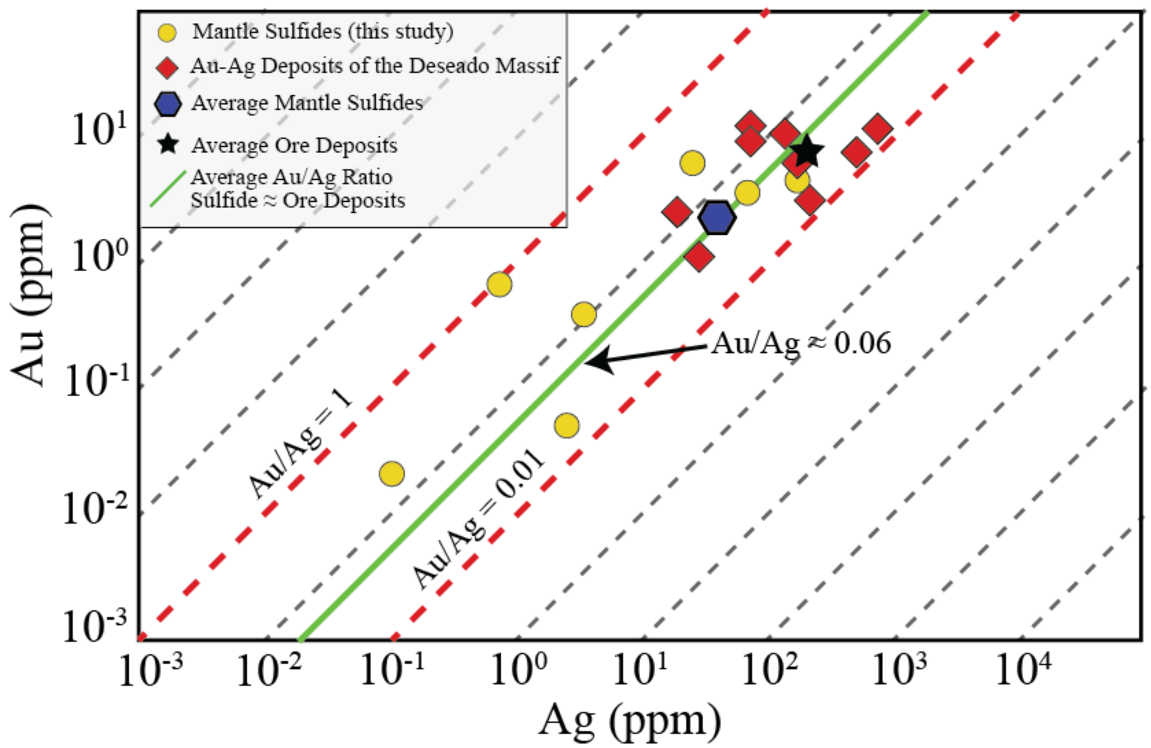

Supplementary Figure 2. Au versus Ag contents of mantle sulphides and epithermal Au-Ag deposits of the Deseado Massif. Dotted lines are constant Au/Ag ratios. Mantle sulphide values from this study (Supplementary Table 3). Epithermal deposits Au-Ag grades taken from refs. 7-13.

Supplementary Figure 2 shows the Au/Ag ratios of mantle sulphides in the late infiltrating melt (glassy vein), and the bulk Au/Ag ratios of the epithermal Au-Ag deposits in the Deseado Massif.

Overall, the bulk Au/Ag ratios of the epithermal deposits in the Deseado Massif are between 0.01 and 0.18, while the Au/Ag ratios of the mantle sulphides vary from 0.02 to 0.95. Thus, the Au/Ag ratios of the epithermal deposits and the sulphides are similar within two orders of magnitude at the most.

Supplementary figure 2 points out that the mantle sulphides may exert an important control on the economic metal ratios of epithermal ore deposits of the Deseado Massif.

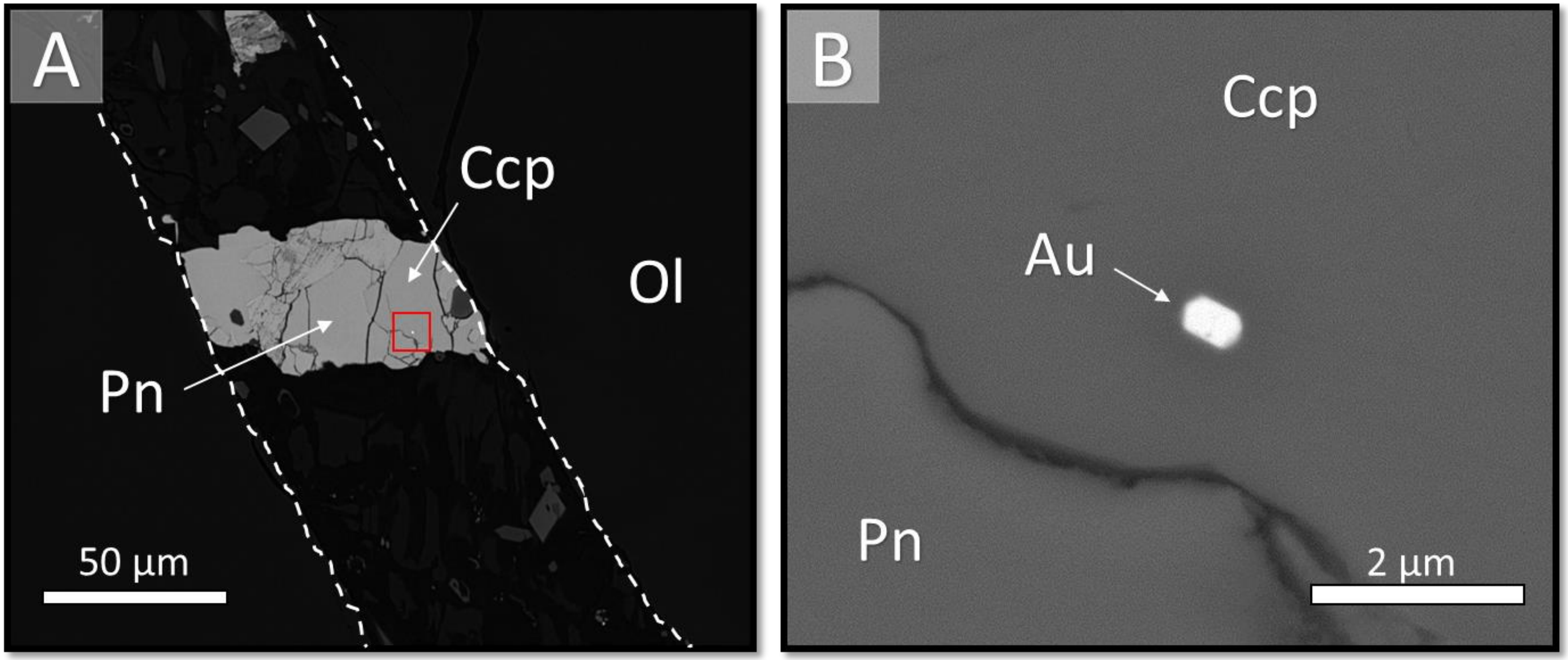

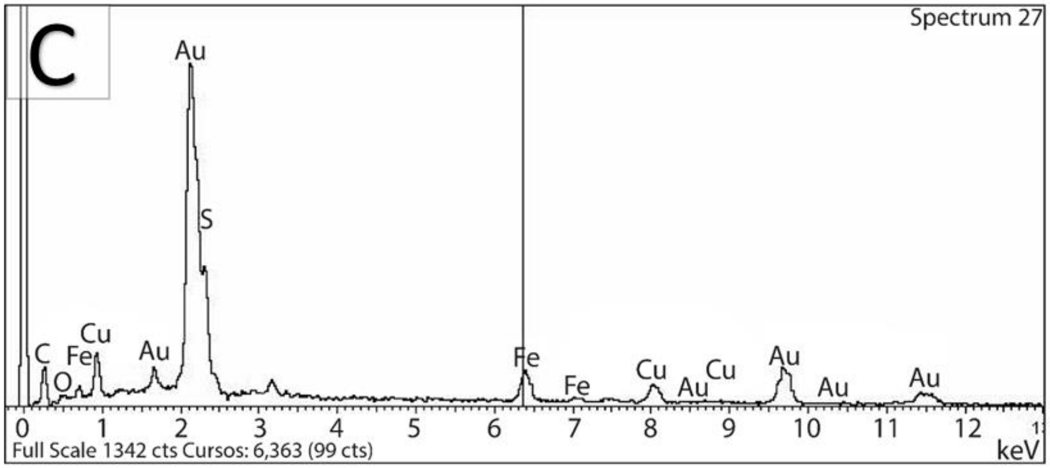

Supplementary Figure 3. FE-SEM backscattered electron images of native gold included within sulphide. A- Composite sulphide in a late metasomatic vein. B- Euhedral gold particle enclosed in the chalcopyrite. C- EDS spectrum of gold particle in “B”.

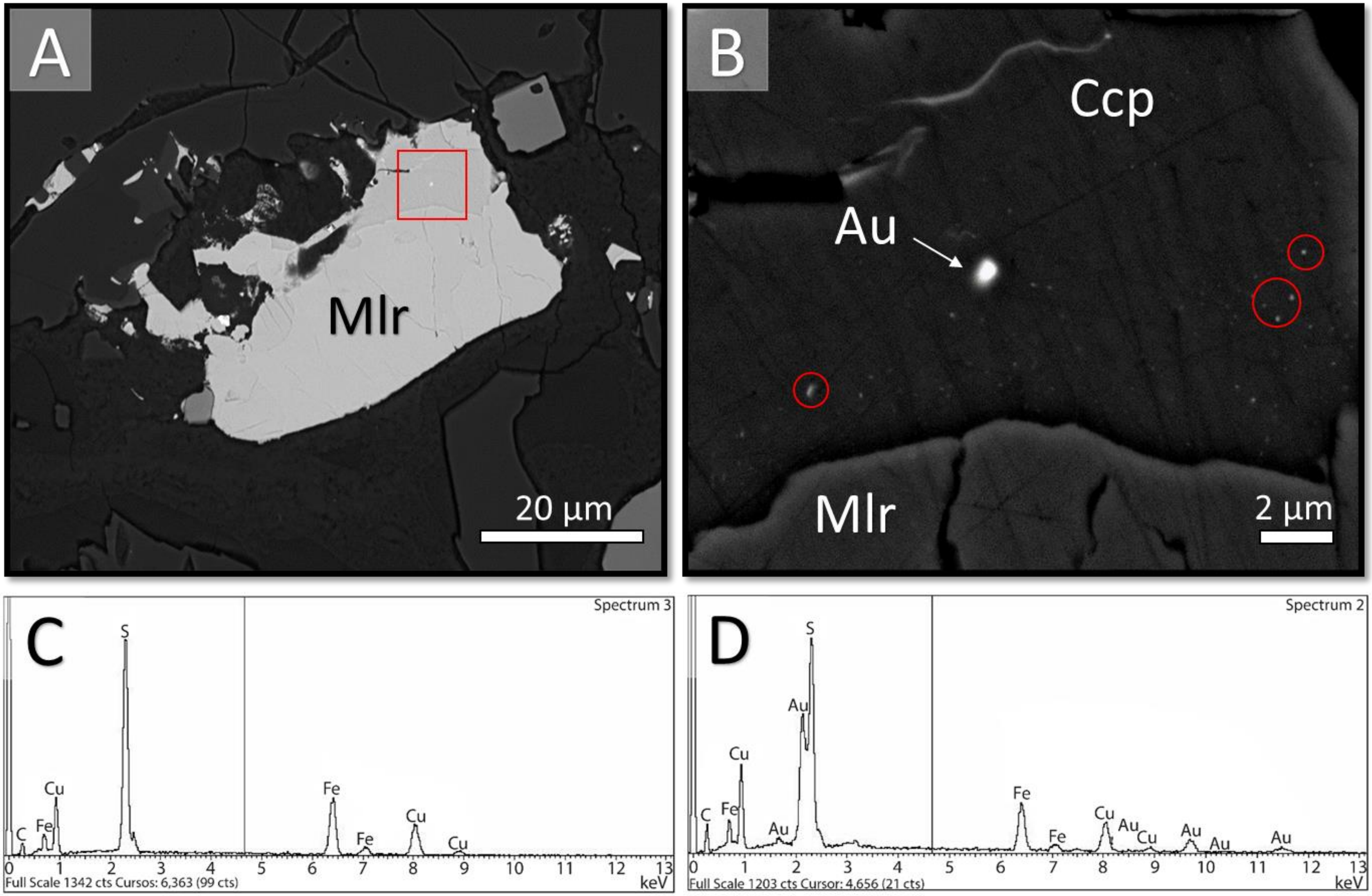

Supplementary Figure 4. FE-SEM backscattered electron images of gold included within sulphide. A- Composite sulphide containing gold particles embedded in a glassy groundmass. B- The inset (red square) in "A" shows a native gold particle (centre) and arrangement of gold nanoparticles within chalcopyrite. C, D- EDS spectrum of the gold particle and surrounding matrix respectively.

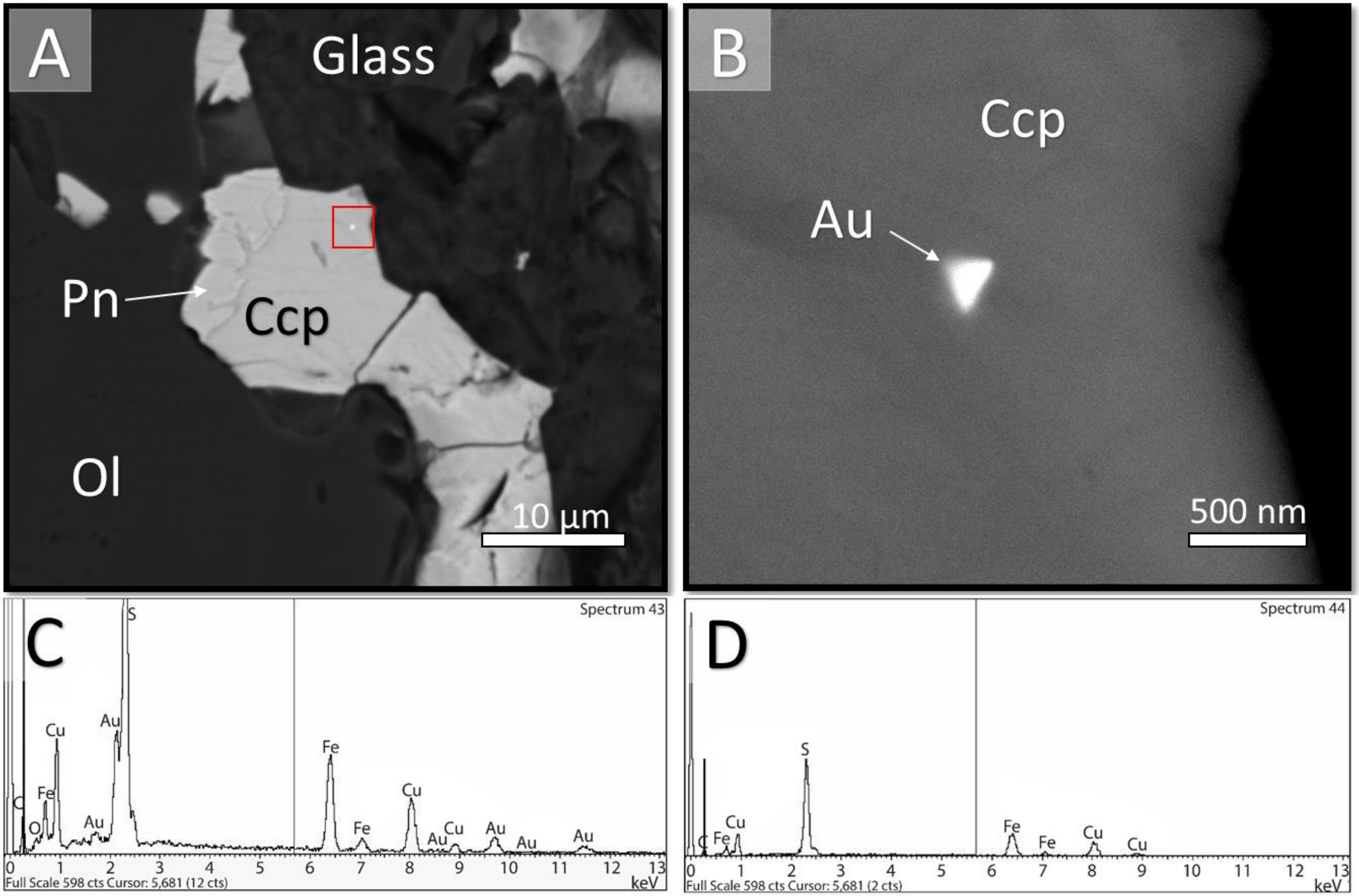

Supplementary Figure 5. FE-SEM backscattered electron images of gold included within sulphide. A- Composite sulphide grain enclosed within devitrified glass in contact with host silicate. B- The inset (red square) in "A" shows a triangular native gold particle enclosed within the chalcopyrite. C,D- EDS spectrum of gold particle in "B" and surrounding matrix respectively.

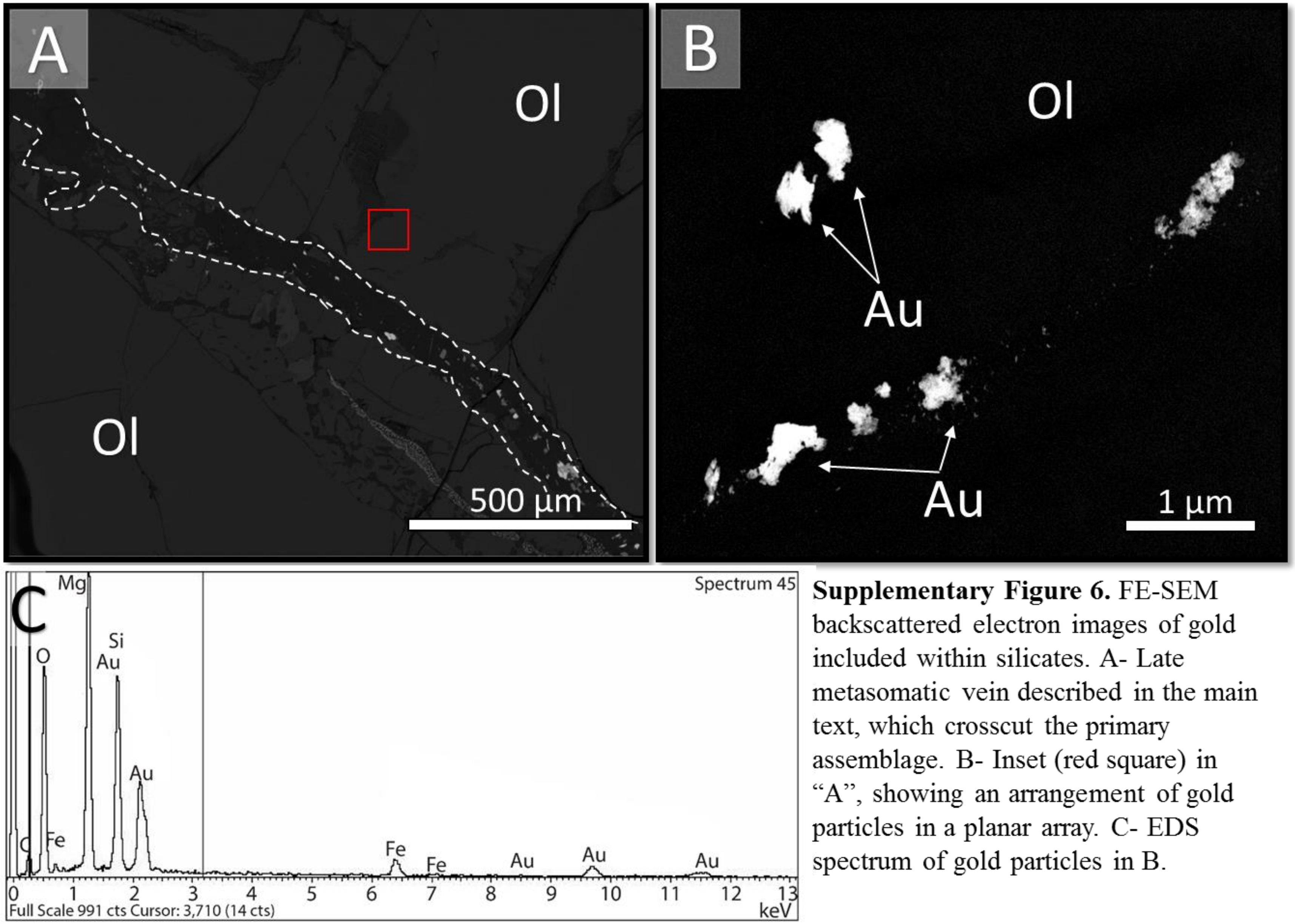

Supplementary Figure 6. FE-SEM backscattered electron images of gold included within silicates. A- Late metasomatic vein described in the main text, which crosscut the primary assemblage. B- Inset (red square) in "A", showing an arrangement of gold particles in a planar array. C- EDS spectrum of gold particles in B.

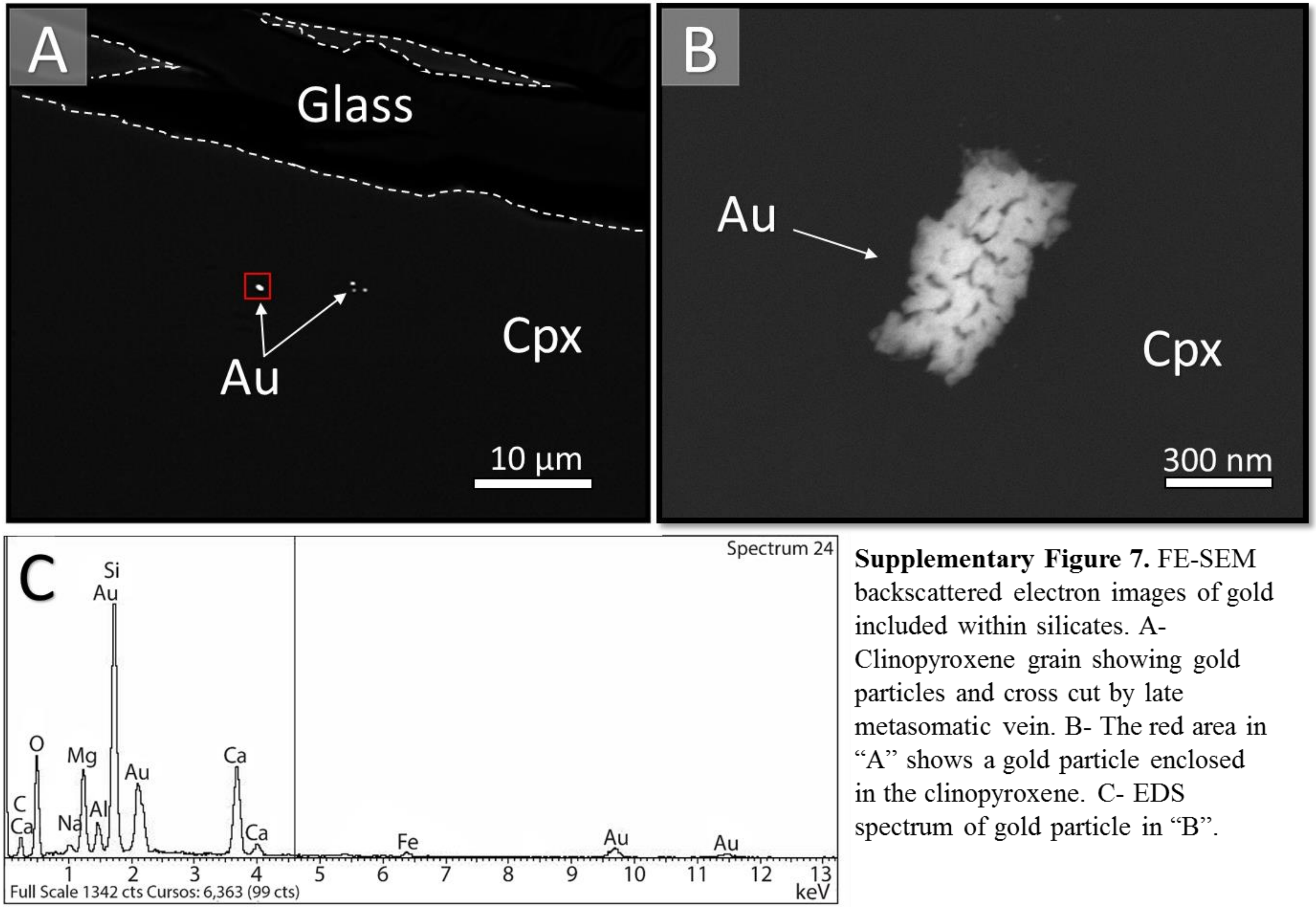

Supplementary Figure 7. FE-SEM backscattered electron images of gold included within silicates. A- Clinopyroxene grain showing gold particles and cross cut by late metasomatic vein. B- The red area in "A" shows a gold particle enclosed in the clinopyroxene. C- EDS spectrum of gold particle in "B".

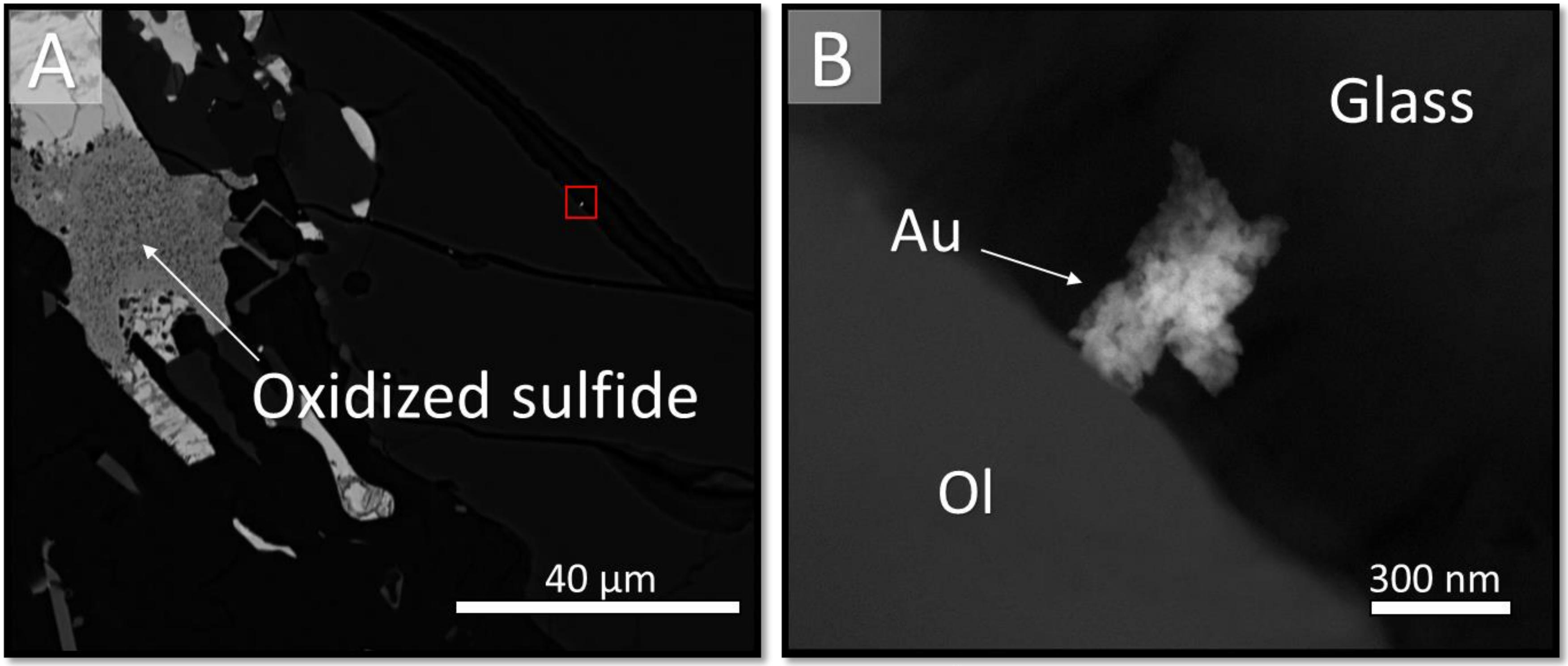

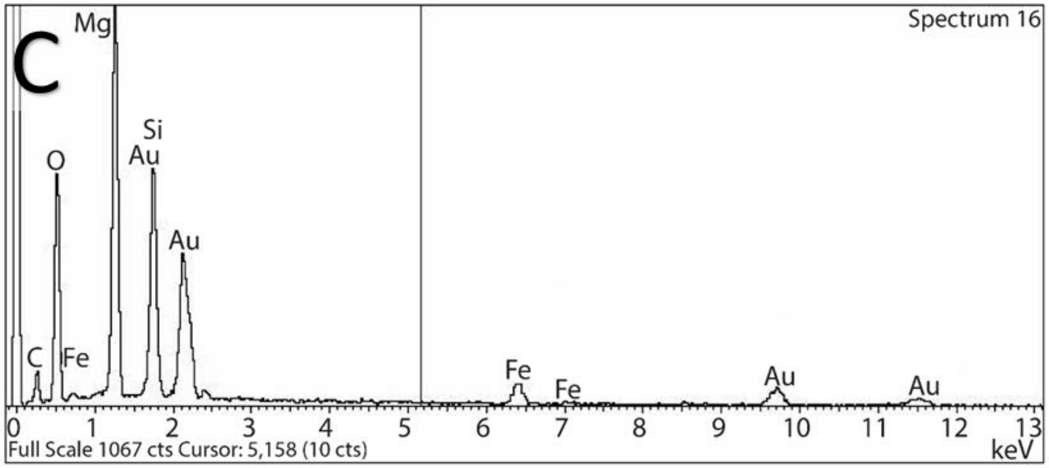

Supplementary Figure 8. FE-SEM backscattered electron images of gold particles included within glass. A- Silicates cross cut by late metasomatic vein containing a partially oxidized sulphide. B- Red square in “A” shows a gold particle within the late introduced glass. C- EDS spectrum of gold particle in B.

Supplementary References

1. Wood, B.J., & Banno, S. Garnet-orthopyroxene and orthopyroxene-clinopyroxene relationships in simple and complex systems. *Contributions to Mineralogy and Petrology* **42**, 109-124 (1973).
2. Brey, G.P. & Köhler, T. Geothermobarometry in Four-phase Lherzolites II. New thermobarometers, and practical assessment of existing thermobarometers. *Journal of Petrology* **35**, 1353-1378 (1990).
3. Putirka, K., 2008, Thermometers and barometers for volcanic systems. *Minerals, Inclusions and Volcanic Processes, Reviews in Mineralogy and Geochemistry, Mineralogical Society of America* **69**, 61-120 (2008).
4. Nell, J., & Wood, B.J. High-temperature electrical measurements and thermodynamic properties of Fe_3O_4-$FeCr_2O_4$-$MgCr_2O_4$-$FeAl_2O_4$ spinels. *American Mineralogist* **76**, 406-426 (1991).
5. Wood, B.J., Bryndiza, L.T. & Johnson, K.E. Mantle oxidation state and its relationship to tectonic environment and fluid speciation. *Science* **238**, 337-345 (1990).
6. McDonough, W.F. & Sun, S. The composition of the Earth. *Chemical Geology* **120**, 223-253 (1995).
7. Schalamuk, I.B., Zubia, M., Genini, A. & Fernandez, R.R. Jurassic epithermal Au_Ag deposits of Patagonia, Argentina. *Ore Geology Reviews* **12**, 173-176 (1997).
8. Echavarría, L.E., Schalamuk, I.B. & Etcheverry, R.O. Geologic and tectonic setting of Deseado Massif epithermal deposits, Argentina, based on El Dorado-Monserrat. *Journal of South American Earth Sciences* **19**, 415-432 (2005).
9. Permuy Vidal, C., Guido, D.M., Moreira, P., Ríos, F.J., Melgarejo, J.C. Características metalogenéticas de Eureka West, principal clavo mineralizado de la veta Eureka, Distrito Cerro Negro, Macizo del Deseado. *Revista de la Asociación Geológica Argentina* **73**, 64-77 (2016).
10. McEwen Mining Inc. San José Mine – Argentina. Resources and Reserves. (Accessed May 20, 2016, at http://www.mcewenmining.com/Operations/Reserves-and-Resources/default.aspx).
11. Goldcorp Inc. Reserves and Resources Notes 2016. (Available at http://s1.q4cdn.com/038672619/files/doc_downloads/2017/Reserves_Resources_Notes_2016.pdf).
12. Patagonia Gold PLC. Projects, Reserves and Resources. (Accessed May 20, 2016, at http://www.patagoniagold.com/projects/reserves-and-resources/).

13. Yamana Gold Inc. 2016 Mineral Resources. (Accessed May 20, 2016, at http://www.yamana.com/English/portfolio/reserves-and-resources/default.aspx).
