# Supplementary material for: Plume-subduction interaction forms large auriferous provinces
Source: Nat Commun. 2017 Oct 10;8:843. doi: 10.1038/s41467-017-00821-z (PMC5634996; doi:10.1038/s41467-017-00821-z)
Supplement: Supplementary file 2 — Data Set 1 [file 41467_2017_821_MOESM2_ESM.pdf]

Reviewers' Comments:

Reviewer #1:
Remarks to the Author:
Dear authors

I enjoyed reading your paper. But consider the following:

I disagree with this; the CA-LIP (a siliceous LIP, SLIP) associated with the Karoo-Ferrar LIP pulses has ages of ~180-185 Ma. Subsequent ages of volcanism pulses in the area are 162-172 Ma and 153-162 Ma. These have calc-alkaline affinity and likely related to subduction processes from the Paficic-Andean margin, as actually suggested by the authors. But as such, these last two pulses are not part of the CA-SLIP. They are subduction related consistent with the Andean setting. The rift system shown in Stage B of figure 2, is probably related to some form of back-arc extension, which affected the overlying CA-SLIP units. And yes, slab-derived fluids triggered melting of an Au-enriched SCLM. But nothing to do with the Chon-Aike mantle plume. Thus, stage B in figure 2, shows a typical subduction system and if there was a rift setting affecting the older CA-SLIP, consideration should be given to a possible back-arc extension and/or slab roll back, rather than an interplay of a mantle plume with subduction-related fluids. The CA-SLIP-Karro-Ferrar mantle plume, was no longer active at the time of the Andean subduction.
Of course, the above observations, which the authors should consider, does not affect the role of native gold being take up in the xenolilth examined (just out of curiosity, can the xenolith examined be dated?).

On the basis of the above concern, I suggest a minor revision

Reviewer #3:
Remarks to the Author:
This is an interesting contribution that definitely merits publication because it addresses a topic of considerable academic and economic interest: the global-scale localisation of relatively restricted blocks of continental crust highly endowed with gold deposits. Furthermore, the detailed documentation of native gold in a mantle xenolith is, in itself, of considerable interest. Prior to acceptance, however, two geological aspects of the study are thought to require further consideration.

(1) The gold deposits of the Deseado massif, in common with those in many gold provinces worldwide, actually contain far more silver than gold. Indeed, as noted again below, Mina Martha is a silver deposit containing little gold. Hence, the lack of any silver in the glassy infiltration veins needs to be explained. And, given the absence of silver in the glassy infiltration veins, where does all the silver in the Deseado deposits come from? The gold and silver in the epithermal deposits are, of course, intimately associated, in part as the alloy electrum.

(2) Fig. 3 and corresponding text: It strikes me that there’s a potential problem here. The assumption seems to be that the mantle plume fertilized the SCLM at ~180 Ma and that later (~155 Ma) subduction caused the gold release. However, Chon Aike magmatism in Patagonia migrated from NE to SW (see ref. 12), eventually generating the Tobífera Formation farther west in Chile. The early plume-dominated stage did not affect the western part of the Deseado massif, where there is no evidence for 180-Ma magmatism, so how could it have fertilized the SCLM directly beneath many of the gold deposits? I suppose it must be assumed that that the 180-Ma event introduced gold to the SCLM but lacked surface manifestations nearby the gold deposits?

The manuscript ends (lines 246-9) by stating that a similar geotectonic scenario could explain the epithermal gold province related to the northern Nevada rift. However, it is well documented (e.g.

Dave John's work) that the northern Nevada rift, formed during early stages of Yellowstone hot-spot activity, did not undergo any subsequent subduction-related magmatism. The magmatic arc was located far to the west along the southern extension of the present-day Cascades. Hence, the two-stage model proposed for the Deseado massif (plume fertilisation followed by subduction-induced gold release) cannot be applied to the northern Nevada rift.

The text needs to be improved to meet the standards of Nature. There are numerous grammatical errors (the worst noted below) that need to be cleaned up by the authors who have English as a first language. The text uses largely (but not exclusively) American spelling and punctuation, which I assume will require conversion to the British equivalent. Elements are written in full or as symbols; standardisation is recommended. In the references, some journal titles are abbreviated whereas most are not; need to follow journal guidelines.

Minor points for attention:

64 should read 'compositions'

66 should read 'remains'

69 These figures need a cited source.

77 Reads poorly. Would suggest 'low sulfidation, intermediate sulfidation and polymetallic….'

83 Should this read 'migration of magmatism'? Difficult to understand as is.

93 Martha Mine (which should read Mina Martha: its formal name) is irrelevant because it is a silver deposit with little gold and your study found no evidence of silver. La Rosita and La Sarita are not Au deposits; they are prospects or occurrences.

127-8 Needs re-writing to make grammatical sense.

136-8 Also grammatically incorrect.

174 re-equilibration??

224-6 Although you use the word 'clearly', the argument is unclear to this reviewer. Please rephrase.

231-3 This sentence needs citations because the conclusion has been stated by others; e.g. Pankhurst and co-workers.

245-6 Where are these supposed trans-lithospheric faults? The only faults mapped are those bounding NNW- and NW-striking half-grabens. The epithermal veins are most likely controlled by volcanic domes emplaced during caldera volcanism.

276-7 Needs rephrasing; currently grammatically incorrect.

315, 317 & 333 should read Antarctic

364 'Acta' missed out

R. H. Sillitoe
17-4-17

On the following pages, we present a response to the reviewers. In this revised version, we have addressed *all* of the comments and suggestions that the reviewers considered most relevant in their report. In addition, we have incorporated most of the changes suggested by the two reviewers (Richard Sillitoe and one anonymous reviewer). For each topic (quote) considered most relevant by each referee, we address the incorporated changes in both science and style (blue).

Author´s response to the specific comments made by reviewers

REVIEWER #1 (anonymous)

Summary & corrections made: This review was very positive and constructive and raised some questions related to the geological background that were not clear enough in the original submission. Based on the reviewer's assessment, we have carefully revised the manuscript and incorporated changes aimed at clarifying these issues, including a slight modification of Figure 3 (Stage B).

R1 QUOTE (1)

"I disagree with this; the CA-LIP (a siliceous LIP, SLIP) associated with the Karoo-Ferrar LIP pulses has ages of ~180-185 Ma. Subsequent ages of volcanism pulses in the area are 162-172 Ma and 153-162 Ma. These have calc-alkaline affinity and likely related to subduction processes from the Paficic-Andean margin, as actually suggested by the authors. But as such, these last two pulses are not part of the CA-SLIP. They are subduction related consistent with the Andean setting. The rift system shown in Stage B of figure 2, is probably related to some form of back-arc extension, which affected the overlying CA-SLIP units. And yes, slab-derived fluids triggered melting of an Au-enriched SCLM. But nothing to do with the Chon-Aike mantle plume. Thus, stage B in figure 2, shows a typical subduction system and if there was a rift setting affecting the older CA-SLIP, consideration should be given to a possible back-arc extension and/or slab roll back, rather than an interplay of a mantle plume with subduction-related fluids. The CA-SLIP-Karro-Ferrar mantle plume, was no longer active at the time of the Andean subduction. Of course, the above observations, which the authors should consider, does not affect the role of native gold being take up in the xenolilth examined".

REPLY TO R1 QUOTE (1)

The reviewer pointed out correctly that the Chon Aike province is a Silicic Igneous province that should be named CA-SLIP instead of CA-LIP, and that is linked with the Karoo-Ferrar flood basalt provinces (refs. 12 and 13). We fully agree with this and we have corrected the text accordingly.

The reviewer main concern relates to the fact that the CA-SLIP should only include the early tholeiitic stages of the magmatism consisting of the pulses of ages ~180-185 Ma.

Although we understand the reviewer's concern, this is not entirely correct. According to most authors (refs. 12, 13; Pankhurst et al., 1998), the CA-SLIP encompasses *all* the extensive volcanism that occurred in Patagonia for ~35 Ma (between 188 and 153 Ma) and is composed of *three* main volcanogenic pulses. The first of these pulses (V1, Early Jurassic, 188-178 Ma) records within-plate affinities and is coincident with the extensive emplacement of mafic igneous rocks in Gondwana generally associated with the Karoo mantle plume. Indeed, the two subsequent pulses of volcanism (V2 and V3, 172-162 and 157-153 Ma respectively) represent a shift towards calc-alkaline affinities and as such, are definitely most likely related to a subduction zone but still part of the extensive loosely defined Chon Aike Province (Pankhurst et al., 1998; ref. 13).

The epithermal Au-Ag deposits of the Deseado Massif are related to the final calc-alkaline stages of the CA-SLIP (e.g., ref. 11). Within this framework, our model includes a precursor stage of mantle plume activity (related to V1) that contribute gold to the SCLM followed by a subduction phase that contributes fluids to trigger the melting of the formerly gold-enriched SCLM (related to pulses V2 and V3). Certainly, the Stage B in Figure 3 shows a subduction system and back-arc setting. Accordingly, we emphasise that the plume-subduction interplay is not necessarily coetaneous. Instead, plume enrichment event might be followed in time by the back-arc subduction setting as a consequence of migration away from the Karoo plume centre.

Based on the reviewer's comment, we have rewritten some parts of the original manuscript (Lines 79-84) in order to avoid confusion and we also slightly modified the Stage B in Figure 3 by removing the "plume feeding finger", as it is not relevant for the model and could induce some misunderstanding.

R1 QUOTE (2)

"(just out of curiosity, can the xenolith examined be dated?)".

REPLY TO R1 QUOTE (2)

To date, the best approach available to estimate the age of the magmatic processes recorded in our xenolith is to determine the Re-Os isotopic composition of individual sulphide grains and calculate their Os model ages (T_{MA}). However, single sulphide grains could also yield inherited $^{187}Os/^{188}Os$ isotopic composition, which would result in model ages much older than the true crystallisation age, even assuming that no Re was present in the sample (i.e., rhenium-depletion age T_{RD}). Therefore, even in the best analytical scenario we probably would not be dating the time of crystallisation of gold-bearing sulphides.

<u>REVIEWER #3 (R. H. Sillitoe)</u>

Summary & corrections made: Richard Sillitoe provided a very helpful and thoughtful review of our manuscript. We have used his report to carefully revise the original submission

and to improve some key aspects of the proposed model. His revision is highly acknowledged.

R3 QUOTE (1)

"The gold deposits of the Deseado massif, in common with those in many gold provinces worldwide, actually contain far more silver than gold. Indeed, as noted again below, Mina Martha is a silver deposit containing little gold. Hence, the lack of any silver in the glassy infiltration veins needs to be explained. And, given the absence of silver in the glassy infiltration veins, where does all the silver in the Deseado deposits come from? The gold and silver in the epithermal deposits are, of course, intimately associated, in part as the alloy electrum".

REPLY TO R3 QUOTE (1)

Richard Sillitoe raised an important point here. The interstitial glassy vein infiltrating the xenolith contains a mineral assemblage that includes armalcolite, ilmenite, feldspar, apatite and sulphide containing native Au particles. We have argued that upon ascent, the infiltrating melt should undergo decompression as well as fractionation and oxidation by reaction with the country rock and that these processes shifted the sulphur balance towards more soluble sulphate, promoting sulphide resorption. The later process would result in the liberation of native Au particles from the sulphides with relatively high contents of Au. Thus, the fact that the native Au particles do not contain Ag does not mean that there is no Ag in the interstitial glassy vein. Instead, as noted in the supplementary table S5, the analysed sulphides in the glassy vein have significant amounts of Ag (up to 163 ppm) as obtained by LA-ICP-MS analyses. We have explicitly cited these data in the text (lines 125-129).

In order to further clarify this issue, we have compared the bulk Au/Ag ratios of the epithermal deposits of the Deseado Massif with those of the sulphides analysed using LA-ICP-MS (supplementary table S5). This is presented as a new figure in the supplementary information (Figure S2) that shows the Au versus Ag concentrations in mantle sulphides and bulk Au-Ag deposits from the Deseado Massif. The bulk Au/Ag ratios of the epithermal Au-Ag deposits are between 0.01 and 0.18 while those of the sulphides are between 0.02 and 0.95. Noticeably, the Au and Ag contents as well as the Au/Ag ratio of the most enriched mantle sulphides plot within the field defined for the Au-Ag deposits of the Deseado Massif. Also, the Au/Ag ratio of the average Au and Ag concentration in the mantle sulphides and the ore deposits are coincident (~0.06, excluding Mina Martha that is a Ag-rich endmember or outlier).

We argue that both Au and Ag are derived from an enriched subcontinental lithospheric mantle source and that the Au/Ag content of the mantle sulphides may exert an important control on the economic metal ratios of Au-Ag epithermal ore deposits of the Deseado Massif.

For the case of the Ag-rich Mina Martha deposit (up to 3500 ppm Ag), where the Au/Ag ratios are much lower (~0.001), we assume that local magmatic-hydrothermal

processes operating at shallower crustal levels favoured the preferential fractionation of Ag besides Au (e.g., Zajacz et al., 2008).

R3 QUOTE (2)

"Figure 3 and corresponding text: It strikes me that there's a potential problem here. The assumption seems to be that the mantle plume fertilised the SCLM at ~180 Ma and that later (~155 Ma) subduction caused the gold release. However, Chon Aike magmatism in Patagonia migrated from NE to SW (see ref. 12), eventually generating the Tobífera Formation farther west in Chile. The early plume-dominated stage did not affect the western part of the Deseado massif, where there is no evidence for 180-Ma magmatism, so how could it have fertilized the SCLM directly beneath many of the gold deposits? I suppose it must be assumed that that the 180-Ma event introduced gold to the SCLM but lacked surface manifestations nearby the gold deposits?"

REPLY TO R3 QUOTE (2)

Certainly, the Chon Aike magmatism migrated from NE to SW as it evolved from tholeiitic affinity in its early stages (~180 Ma) to calc-alkaline affinity (~155 Ma) in its late stages. Indeed, in our model, the mantle plume fertilised the SCLM at ~180 Ma and the later (~155 Ma) subduction caused the gold release.

The observation made by the reviewer that there is no ~180 Ma magmatism expression in the western Deseado Massif is correct. However, the ~180 Ma tholeiitic magmatism has a large areal distribution since it can be found from the north-eastern Patagonia, to the eastern and southern Deseado Massif itself, Tierra del Fuego in the southernmost Patagonia, and even in the Antarctic Peninsula (see ref. 13). This means that the ~180 Ma magmatism crops out irregularly along far more than a thousand kilometres. Based on these observations, we argue that it can be assumed that the plume activity (represented by the ~180 Ma magmatism) also had its enrichment effect in the SCLM beneath the western parts of the Deseado Massif even if there is no ~180 Ma magmatism, as the effect of the mantle plume activity in the deep lithosphere should be larger than its surficial expression. We agree with the reviewer that some of these volcanic units may lack surface manifestations nearby the gold deposits.

R3 QUOTE (3)

"The manuscript ends (lines 246-9) by stating that a similar geotectonic scenario could explain the epithermal gold province related to the northern Nevada rift. However, it is well documented (e.g. Dave John's work) that the northern Nevada rift, formed during early stages of Yellowstone hot-spot activity, did not undergo any subsequent subduction-related magmatism. The magmatic arc was located far to the west along the southern extension of the present-day Cascades. Hence, the two-stage model proposed for the Deseado Massif (plume fertilisation followed by subduction-induced gold release) cannot be applied to the northern Nevada rift".

REPLY TO R3 QUOTE (3)

We agree. This paragraph has been eliminated.

R3 QUOTE (4)

The text needs to be improved to meet the standards of Nature. There are numerous grammatical errors (the worst noted below) that need to be cleaned up by the authors who have English as a first language. The text uses largely (but not exclusively) American spelling and punctuation, which I assume will require conversion to the British equivalent. Elements are written in full or as symbols; standardisation is recommended. In the references, some journal titles are abbreviated whereas most are not; need to follow journal guidelines.

REPLY TO R3 QUOTE (3)

The manuscript has been revised and standardised to British spelling and punctuation. The references are not abbreviated now.

REVIEWER #3 MINOR POINTS FOR ATTENTION:

- 64 should read ‘compositions’
  - Fixed (now line 65).
- 66 should read ‘remains’
  - Fixed (now line 67).
- 69 These figures need a cited source.
  - Agreed. We provide the following references: Schalamuk et al., 1997; Sillitoe et al., 2008; Permuy Vidal et al., 2016; Technical reports of mining companies (in supplementary information) (now line 70).
- 77 Reads poorly. Would suggest ‘low sulfidation, intermediate sulfidation and polymetallic….’
  - Agreed and corrected (now line 78)
- 83 Should this read ‘migration of magmatism’? Difficult to understand as is.
  - Accepted (now line 86).
- 93 Martha Mine (which should read Mina Martha: its formal name) is irrelevant because it is a silver deposit with little gold and your study found no evidence of silver. La Rosita and La Sarita are not Au deposits; they are prospects or occurrences.
  - We have modified this. Mina Martha was removed. (Now line 97).
- 127-8 Needs re-writing to make grammatical sense.
  - Agreed, we have rewritten this (now line 135-6).
- 136-8 Also grammatically incorrect.
  - Agreed, rewritten (now line 144-7).
- 174 re-equilibration??
  - Agreed, corrected (now line 182).
- 224-6 Although you use the word ‘clearly’, the argument is unclear to this reviewer. Please rephrase.

- Agreed, rewritten (now line 233-5).

- 231-3 This sentence needs citations because the conclusion has been stated by others; e.g. Pankhurst and co-workers.
  - OK, cited (now line 243).
- 245-6 Where are these supposed trans-lithospheric faults? The only faults mapped are those bounding NNW- and NW-striking half-grabens. The epithermal veins are most likely controlled by volcanic domes emplaced during caldera volcanism.
  - Agreed, rewritten (now line 254-5).
- 276-7 Needs rephrasing; currently grammatically incorrect.
  - Agreed, rewritten (now line 278-279).
- 315, 317 & 333 should read Antarctic
  - OK, corrected.
- 364 ‘Acta’ missed out
  - OK, corrected.
  -

References used in the response to the reviewers (not in the manuscript)

Pankhurst, R., Leat, P.T., Sruoga, P., Rapela, C.W., Márquez, M., Storey, B.C. & Riley, T.R. The Chon Aike province of Patagonia and related rocks in West Antarctica: A silicic large igneous province. *Journal of Volcanology and Geothermal Research* **81**, 113-136 (1998).

Zajacz, Z., Halter, W.E., Pettke, T. & Guillong, M. Determination of fluid/melt partition coefficients by LA-ICPMS analysis of co-existing fluid and silicate melt inclusions: controls on element partitioning. *Geochimica et Cosmochimica Acta* **72**, 2169-2197 (2008).

Reviewers' Comments:

Reviewer #3:
Remarks to the Author:
The authors have already incorporated my recommendations (and those of other reviewers) in their revised manuscript. I was happy with their response to my commentary.

Author´s response to the specific comments made by reviewers

REVIEWER #3 (R. H. Sillitoe)

(Remarks to the Author)
R1 QUOTE (1)

The authors have already incorporated my recommendations (and those of other reviewers) in their revised manuscript. I was happy with their response to my commentary.

REPLY TO R1 QUOTE (1)

Thank you.
